# The resistive ground fault of PWM voltage inverter in the EV charging station

**DOI:** 10.1038/s41598-021-00715-7

**Published:** 2021-10-27

**Authors:** Marta Zurek-Mortka, Jerzy R. Szymanski

**Affiliations:** 1 Department of Control Systems, Lukasiewicz Research Network-Institute for Sustainable Technologies, 26-600 Radom, Poland; 2 Faculty of Transport, Electrical Engineering and Computer Science, Kazimierz Pulaski University of Technology and Humanities, 26-600 Radom, Poland

**Keywords:** Engineering, Electrical and electronic engineering

## Abstract

During the direct touch of the inverter output voltage or with the ungrounded shield of the cable connecting the inverter to the motor or other type of load, the nonsinusoidal ground currents with a basic harmonic frequency between 1.5 and 16 kHz, flow via a human’s body. Here was proved that Residual Current Device (RCD) ($I_{\triangle n}$ = 30 mA) does not switch off the power supply when a ground current with a value of about some hundred milliamps occurs. Because RCDs do not disconnect the power supply, the touch on the inverter’s voltage is dangerous to health and life. For the authors, the RCD usage in the Voltage Frequency Converters (VFCs) is not a good engineer practice when high-frequency common-mode distortion currents flow through it. The paper presents tests of RCD operation in the event of a resistance ground fault (via human body) during EV battery charging where the PWM voltage inverter is connected to the external rectifier to provide DC charging battery voltage. Finally, the authors propose a method of eliminating common-mode (CM) current from short protection system by using a separate circuit in which the parasitic leakage current omits an RCD.

## Introduction

In drive voltage frequency converters (VFCs), the PWM (Pulse Width Modulation) voltage inverter is a DC/AC converter that is usually connected to the motor. The drive VFCs can also be used as a part of EV (electric vehicle) battery charger converters^1^ as an external battery charger. The PWM voltage inverter used in EV to feed the vehicle’s driving motor (synchronous or asynchronous) can also be additionally used as a basic component onboard battery charger.

**Figure 1:**
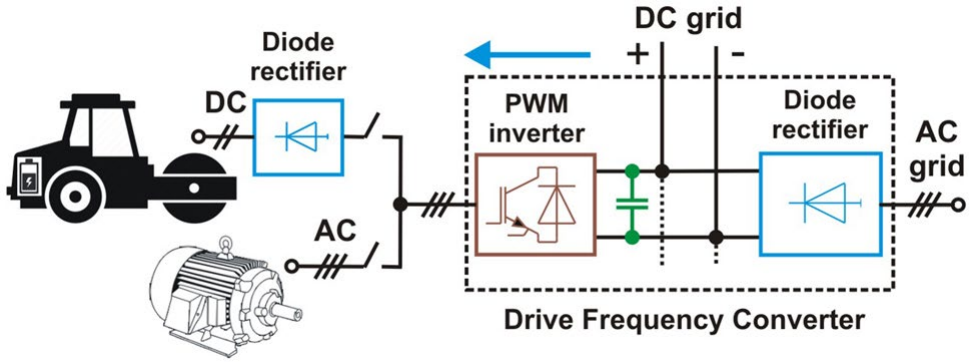
Proposed solution of the double function in the drive VFC^1^.

Figure 1 shows the concept of using a low-voltage (LV) drive VFC for the implementation of two functionalities: control of induction motors and charging of EV batteries and energy storage. The main difference between these two functions is that for motor drives, the PWM drive converter produces a three-phase sinusoidal voltage with a frequency of basic harmonic from 0.5 to 50 Hz/60 Hz, where 50 Hz/60 Hz is usually the frequency of steady-state motor operating. On the other hand, when rectifying the three-phase voltage, the maximum frequency of the basic harmonic is used for battery charging and it changes within a small range, e.g. 250–300 Hz or 900–1000 Hz, depending on the properties of the drive converter. The presented solution is described in detail in^1,2^, other types of converters used in the EV battery charging stations are presented in^3^. The basic harmonics of the phase voltage of PWM inverter reaches the frequency up to kilohertz^4^ (this is inverter switching frequency) when the PWM amplitude modulation factor is near zero. Drives with VFCs are commonly powered by transformers with TN network arrangement^5^. In the event of a resistive ground fault to the phase voltage of the inverter, the resulting short-circuit current has a limited effective value and does not stop the inverter, therefore there is a risk of electric shock. The earthing via the human body has resistance about 1 k$\Omega$. In drive systems with VFCs, the RCDs, regardless of their type and design, must not be sensitive to ground capacitance leakage currents caused by the inverter CM voltage, otherwise, they may prevent the operation of the VFC in short-circuit condition when AC with frequency 50 Hz/60 Hz or constant current occurs^6^. The CM distortion currents caused by CM voltage have a frequency that depends on the switching frequency of the inverter power elements (IGBT, GTO, and others).One method is the usage of CM filters^7^, which are designed to limit the effects of the occurrence of inverter CM voltage on CM disturbances on the motor and cable or to create a path for leakage current flow that bypass the grounded parts of the drive system, in particular, the PE (protective earth) installation and the transformer.

A resistive short-circuit is direct human contact with the inverter phase voltage. Under normal environmental conditions, the human body resistance has a normalized value of 1 k$\Omega$^5,8^. In the event of human shock, the ground fault currents caused by the inverter voltage are not detected by the residual current devices used in drive systems^9,10^. The high-frequency capacitance leakage currents flowing in the electric shock protection system pose a risk of electric shock.

The safe operation of an EV battery charging station depends on the presence of appropriate protections on the supply side, including a fuse or an overcurrent circuit breaker, correct connection to the ground cables screens and the components metallic chassis also right usage RCD for disconnecting the power supply if the ground leakage current or ground fault current is greater than a specified value (e.g. 30 mA). The problem of the lack of adequate protection concerns EV batteries in mode 1, where the EV is connected to the AC network through a standardized socket with currents up to 16 A, is described in^11,12^. In^13^ the method of detecting ground faults in modern electrical installations is described, especially with the use of a drive VFC. In^14^ the tests of the sensitivity of the type A RCD are presented, showing a differentiated check depending on the harmonic current content. The main conclusion is that so far there are no properly functioning RCDs for protection against electric shock. When using RCDs with VFCs containing a PWM inverter, the author suggests using magnetic material with high efficiency and a narrow hysteresis loop for the construction of the Ferranti coil. This will enable the correct measurement of capacitive ground leakage currents with harmonics with frequencies of the order of kHz (e.g. from 3 to 20 kHz). In^15^, the authors propose a comprehensive solution for an AC charging station for EV batteries in mode 3, which includes a communication system, RCD protection, and an application for controlling the energy flow.

**Figure 2:**
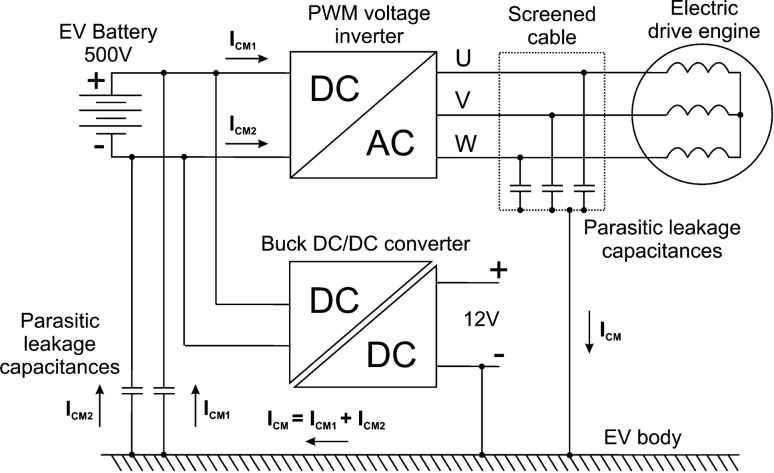
Leakage currents caused by the common-mode voltage of the PWM voltage inverter in the EV drive system.

This work is another attempt to demonstrate the complexity of the problem of using RCDs in the PE protection system of converters with PWM inverter in the EV battery charging systems (onboard or external) or EV motor drive. The power supply system of the EV drive motor is shown in Fig. 2^16^.

A PWM inverter is responsible for the permissible drive torque and the rotational speed of the motor shaft and is powered by a high-voltage vehicle battery. The battery voltages usually do not exceed 500 V, with maximum powers and maximum capacities in electric vehicles being around 150 kW and 150 kWh, respectively. To supply the drive motor with a PWM inverter, its power must be close to that of the motor (Fig. 2), therefore this inverter can also be used to charge the EV battery when the vehicle is parked (in this situation inverter is not used for feed of motor). The power of the onboard inverter allows the battery to be fully charged within one hour. When an inverter is used to drive the motor and charge the battery, it requires special precautions against electric shock and fire hazards. It is necessary to analyze the effects of inverter CM voltage as its inherent feature. The solution shown in Fig. 2 is used a double-shielded motor cable to create an additional circuit for capacitive leakage currents so that these currents do not flow through the grounded elements of the EV. The leakage currents of the motor are assumed to be negligible.

The use of a PWM voltage inverter as an EV battery charger module has not been discussed in the scientific literature so far. The diagram of a battery charger with the use of a drive converter presented in Fig. 1 is a solution published in 2020. Drive converters are commonly used in industrial drives. High-frequency capacitive leakage currents flow through PE protection systems and have not been effectively contained so far. In this paper, the authors showed that RCDs are not an effective solution to protect humans against electric shock. Ground leakage currents with values endangering human safety flow through RCDs under normal operating conditions of PWM voltage inverters and may cause the charger to be powered off by accident and thus prevent its proper operation. According to the authors, RCDs should not be used in these applications if capacitive leakage currents flow through the RCD.

The paper is organized as follows: “Drive system and EV charging station with a voltage frequency converter” describes the design of an EV charging system with a VFC and output external rectifier. “Model of the EV charging system” provides analytical descriptions of CM distortions and the model for simulation tests. “Results” shows the laboratory stand for detection of the ground fault via resistance of the human body. The experimental test was conducted for the following cases: shorting of the phase voltage of the inverter and shorting of the ungrounded screen of the screened three-phase cable which connects the inverter and the rectifier. This experiment confirms the made assumptions and simulation results. The methods of limiting the effect of inverter CM voltage on the ground leakage current are described in “Methods for minimizing the capacitive ground leakage currents”. These methods enable the correct operation of RCDs with a VFC. The concluding remarks are given in “Conclusions”.

## Drive system and EV charging station with a voltage frequency converter

**Figure 3:**
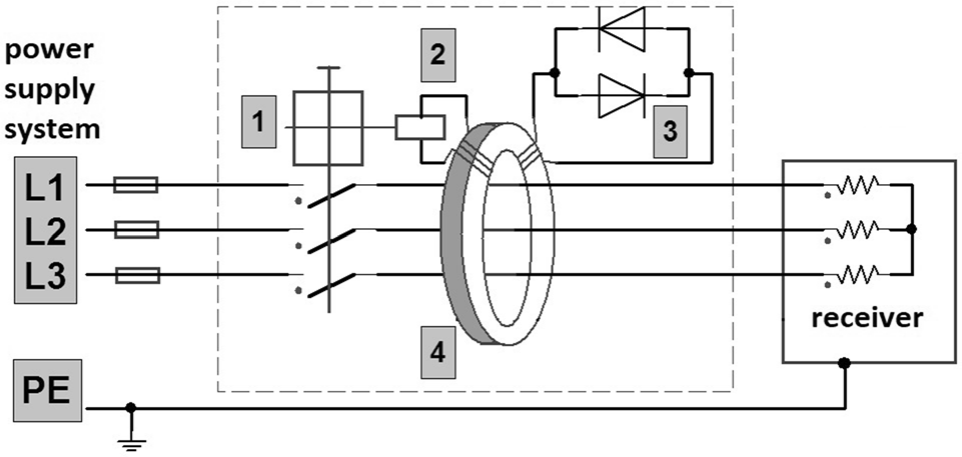
Residual Current Device with a Ferranti coil: 1—lock, 2—differential release, 3—anti-parallel diodes, 4—Ferranti coil^9^.

**Figure 4:**
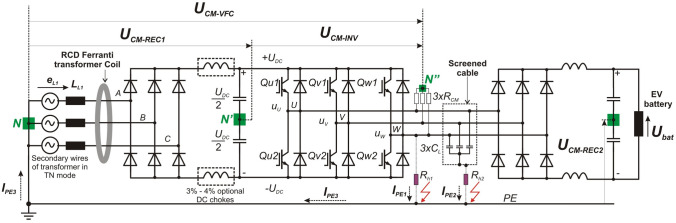
The model of the EV charging system with the VFC and the residual current device for protection against ground fault.

As a protection against a resistive ground fault (human touch), a residual current device with a Ferranti coil was used, the example structure of which is shown in Fig. 3. An EV battery charging converter with a VFC with an RCD on the power supply terminals is shown in Fig. 4. The converter is powered by a transformer with a TN network system. The full ground fault of the inverter output voltages is protected by an electronic circuit that blocks the inverter IGBT switches when the allowable ground current value is exceeded, typically greater than 0.1 nominal current of the inverter. This value is much higher than currently safe for humans. From the side of the power supply rectifier inputs, the VFCs are secured with fast *gR* type fuses, they are device protections and do not react to the occurrence of small currents that occur when people touch them directly.

The resistive ground fault current of the inverter voltage flows via the PE conductor to the transformer, then via the second wires of the transformer to the residual current device (Ferranti coil) to end the current circuit in the inverter. The short-circuit resistance $R_{h}$ (human resistance) has a value of 1 k$\Omega$ and represents the resistance of the human body in direct contact. The short-circuit current is forced by the high-frequency phase voltage of the inverter. In the PWM sinusoidal modulation, the inverter phase voltages $U_{U}$, $U_{V}$, $U_{W}$ have the shape of a square wave with the modulated waveform frequency $f_{c}$ (carrier frequency). The inverter phase voltage frequency $f_{c}$ with IGBT switches is between 2.5 and 16 kHz^4^. When a resistive ground fault occurs, distorted CM currents with the basic harmonic frequency $f_{c}$ flow via the RCD.

Manufacturers of RCDs usually specify their properties for the 50–60 Hz industrial network voltage frequency, while the properties of these devices at frequencies in the order of several kHz are not tested and currents at these frequencies may lead to the accidental shutdown of the protected devices^17,18^. The widespread use of drives with VFCs contributes to the use of RCDs as protection against ground faults.

For the industrial network voltage of 3 $\times$ 400 V/50 Hz, the square wave amplitude $U_{p}$ is equal to 280 V, $\omega _{c}=2\pi f_{c}$ is the pulsation of PWM sinusoidal modulation of the IGBT switches. For industrial drive VFC up to about 100 kW, a carrier frequency $f_{c}$ has typically the ranges from 4 to 5 kHz^4^.

**Figure 5:**
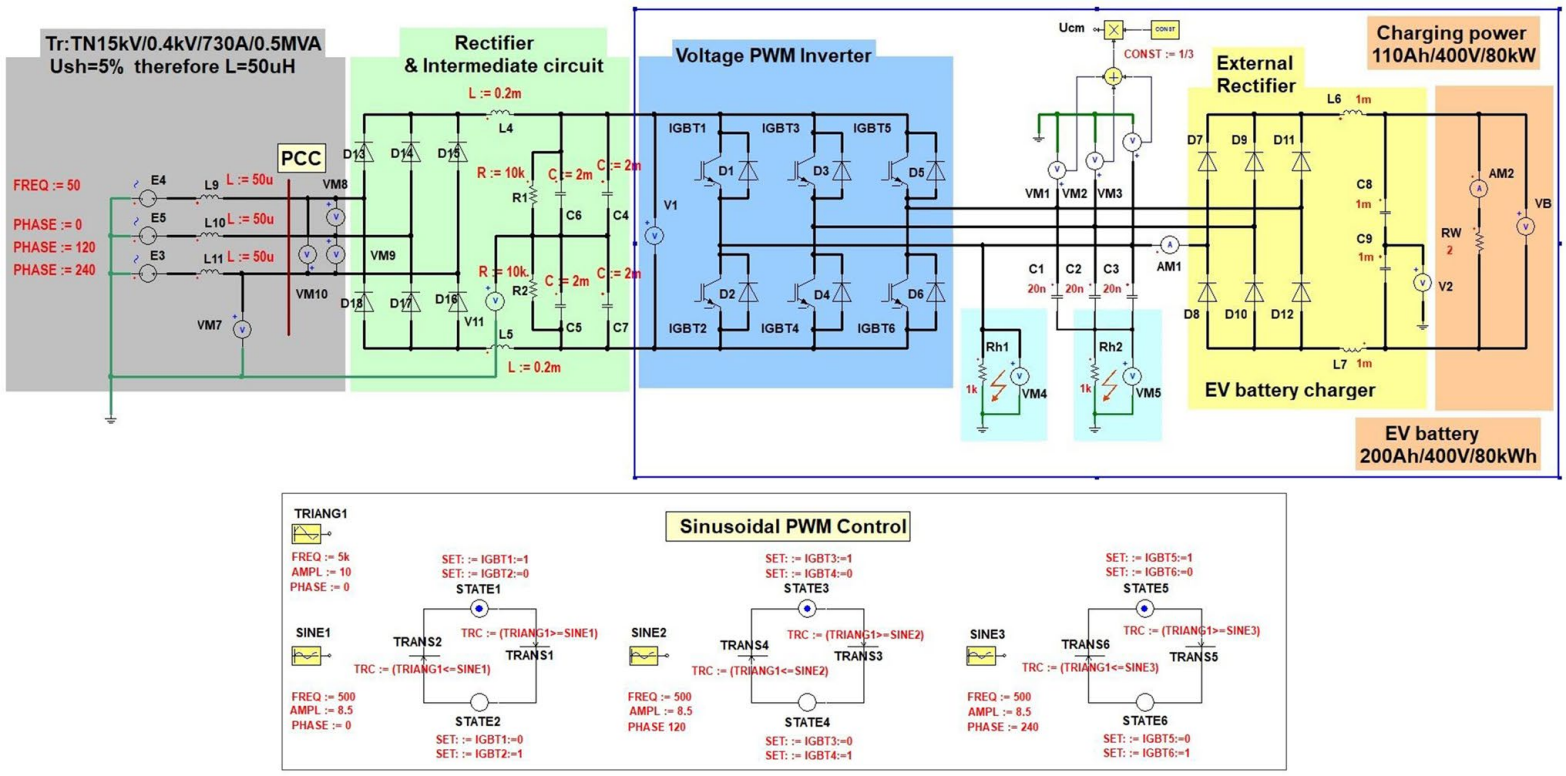
The model of the EV battery charging system with a drive VFC (rectifier and intermediate circuit + voltage PWM inverter) feed by transformer in the TN system.

Equation (3) shows that the ground fault current on $R_{h1}$ (Fig. 4) of the inverter phase voltage will contain odd harmonics and the amplitudes of successive harmonics are quickly suppressed. The basic short-circuit current energy is transferred by the basic harmonic with the frequency of $f_{c}$ = 5 kHz (according to Fig. 5).

In the general case of the converter CM voltage controlling the supply voltage of the rectifier for EV battery charging is described by (1):

1 $$\begin{aligned} U_{CM-VFC}= U_{CM-REC1} + U_{CM-INV} \end{aligned}$$

where:

2 $$\begin{aligned} U_{CM-REC1} = 3U_{DC} \left[ {\frac{1}{{8\pi }}\sin (3\omega t) + \frac{1}{{80\pi }}\sin (9\omega t) + \cdots } \right] \end{aligned}$$

Since the maximum value of rectifier CM voltage $U_{CM-REC1}$ does not exceed 81 V $(\sqrt{2}U_{p}:4)$^19^ and the frequency of this triangular waveform has tripled the supply voltage frequency 3 $\times$ 50 Hz or 3 $\times$ 60 Hz, it is usually ignored in the analysis of ground leakage currents in converters and PWM inverters.

**Figure 6:**
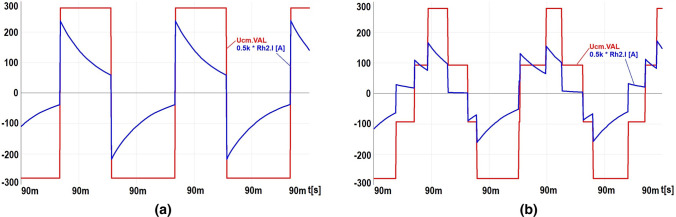
Waveforms of CM voltage and CM ground current with 1 k$\Omega$ resistance for modulation factor: (**a**) $\hbox {M}=0$, (**b**) $\hbox {M}=0.85$.

The inverter phase voltages with the modulation factor M = 0 are rectangular voltages with the same filling (Fig. 6a). There is no phase shift between them and they are described as follow (3):

3 $$\begin{aligned} U_{UN'}=U_{VN'}=U_{WN'}=1.27\cdot U_{p}sin\omega _{c}t+0.424\cdot U_{p}sin3\omega _{c}t+0.255\cdot U_{p}sin5\omega _{c}t+\cdots \end{aligned}$$

where $U_{p}$ is the square pulse amplitude, which is:$\frac{\sqrt{2}U_{ij}}{2}=\frac{U_{DC}}{2}$, where $U_{ij}$ is the phase-to-phase voltage of the transformer feeding of the VFC, where $ij = AB, BC, CA$.

The inverter CM voltage $U_{CM-INV}$ can be determined in two ways:

1. Using the notation of individual voltages written analytically using the Fourier series^6^.
2. Using the vector description of the voltage inverter and the value of the CM voltage assigned to the individual complex (spatial) control vectors of the inverter^20^.

According to the second method, the inverter CM voltage $U_{CM-INV}$ can be determined based on the drive state of the inverter semiconductor valves^20^. This procedure enables the determination of inverter CM voltage value depending on the state of the control vectors of the tested three-phase inverter (Fig. 4).

Table 1 shows the inverter phase voltages and the inverter CM voltage $U_{CM-INV}$ are depending on the state of inverter control vectors $V_0$–$V_7$. The inverter ground fault voltage $U_{CM-INV}$ can be described by (4).

4 $$\begin{aligned} U_{CM-INV} = \left\{ \begin{array}{ll} \pm \frac{{U_{DC} }}{2}&{}\quad for\; vectors\;V_0 \; and \;V_7\;(passive \; vectors) \\ \\ \pm \frac{{U_{DC} }}{6}&{}\quad for\;vectors\; from \;V_1 \; to \;V_6\;(acctive \; vectors) \\ \end{array}\right. \end{aligned}$$

The Equation (3) shows that the harmonics of CM voltage decompose at multiples of the carrier frequency of the PWM modulation (the switching frequency of the inverter IGBTs). Table 1 shows that the inverter CM voltage has a different value depending on the drive state of the IGBT switches (1/6 $\times$ $U_{DC}$ or 1/2 $\times$ $U_{DC}$). The CM voltage waveform with an amplitude of 1/2 $\times$ $U_{DC}$ and frequency $f_c$ of the order of kHz result in a significant electroshock hazard.

**Table 1:** Inverter CM voltage $U_{CM-INV}$ depending on the state of $V_n$ control vectors^20^.

Number [n]	*State*	$U_{UN'}$	$U_{VN'}$	$U_{WN'}$	$U_{CM-INV}$
$V_0$	0, 0, 0	$-U_{DC}$/2	$-U_{DC}$/2	− U_DC_/2	$-U_{DC}$/2
$V_1$	0, 0, 1	$-U_{DC}$/2	$-U_{DC}$/2	− U_DC_/2	$-U_{DC}$/6
$V_2$	0, 1, 1	$-U_{DC}$/2	$U_{DC}$/2	$U_{DC}$/2	$U_{DC}$/6
$V_3$	0, 1, 0	$-U_{DC}$/2	$U_{DC}$/2	− U_DC_/2	$-U_{DC}$/6
$V_4$	1, 1, 0	$U_{DC}$/2	$U_{DC}$/2	− U_DC_/2	$U_{DC}$/6
$V_5$	1, 0, 0	$U_{DC}$/2	$-U_{DC}$/2	− U_DC_/2	$-U_{DC}$/6
$V_6$	1, 0, 1	$U_{DC}$/2	$-U_{DC}$/2	$U_{DC}$/2	$U_{DC}$/6
$V_7$	1, 1, 1	$U_{DC}$/2	$U_{DC}$/2	$U_{DC}$/2	$U_{DC}$/2

## Model of the EV charging system

Figure 5 shows an industrial drive converter model with a voltage inverter, in which the motor is replaced by an external diode rectifier, therefore its functionality has changed and now serves charging function for EV batteries or industrial electric work machines. Since the rectifier CM voltage loaded with a battery of capacitors and supplied from a transformer with a TN network system is negligible, therefore, in the further part of the study, the inverter CM voltage will be only analyzed (frame marked). The RCD is located on the power supply side of the converter (showed as a PCC). The fast *gR* fuses or circuit breakers are also placed there (following the converter manufacturer’s guidelines). The CM leakage current of the PE protective conductor flows into the transformer, distorting the supply voltages and thus disturbing the operation of other receivers connected to the transformer, including RCDs. Typical EMC filters used in drive converters are often not effective enough.

It should be noted that the CM voltage marked on the model as $U_{CM}$ is the CM voltage $U_{CM-INV}$ according to Fig. 4. The inverter CM voltage tests presented in the study relate to the case of powering the inverter from a 560V DC microgrid source, not from the 3 $\times$ 400 V/50 Hz industrial network with a TN transformer. In both cases, the final conclusions about inverter CM voltage effects on RCD are similar.

A linear mathematical model of the EV charging system with an inverter controlled by a sinusoidal PWM modulator was built to conduct simulation tests of the resistive ground fault current of the inverter voltage using ANSYS Twin Builder. The mathematical model has been symbolically written using the wiring diagram shown in Fig. 5. The current–voltage characteristics of the inverter diodes and switches have been linearized. In the inverter model, universal valve characteristics of freewheeling diodes and IGBT switches were adopted. The inverter phase voltage was investigated in the range of continuous sinusoidal modulation^21^, i.e., for the modulation factor from M = 0 to M = 1, where M is defined as (according to the sinusoidal PWM control in Fig. 5) (5):

5 $$\begin{aligned} M=\frac{amplitude~of~sine~modulating~voltage}{amplitude~of~carrier~ wave~(triangular)}=\frac{amplitude~SINE1}{amplitude~TRIANG1} \end{aligned}$$

The PWM voltage inverters produce a three-phase AC voltage, the inherent feature of which is the presence of the voltage on the ground—means the inverter CM voltage, which is described by (according to Fig. 4) (6):

6 $$\begin{aligned} U_{CM-INV}=\frac{1}{3}(U_{N'U}+U_{N'V}+U_{N'W}) \end{aligned}$$

According to the model in Fig. 5, the CM voltage with respect to ground (PE) was determined (1).

The inverter CM voltage has an RMS value greater than 0 (the RMS maximum value of CM voltage reaches $U_{DC}$/2) and forces the flow of high-frequency capacitive leakage current via the converter, power supply system, and PE protection system. When using RCDs for electric shock or fire protection, the capacitive leakage current may cause accidental operation of these switches or their inactivity in the event of an electric shock or fire hazard.

The inverter phase voltage is a square wave with a frequency of 5 kHz (Fig. 5). Due to the constant RMS value of the inverter phase voltage $U_{w}=280V(U_{w}=[\sqrt{2}\cdot 400V]:2)$, independent from the value of PWM modulation factor M, a resistive ground fault ($R_{h1}$) creates shock current with value 280 mA (280 V/1 k$\Omega$). Such a current value is dangerous to humans and will not activate the converter overcurrent protection. This current only can activate RCD.

When touching an ungrounded screen of the cable connecting inverter with the battery charging rectifier ($R_{h2}$), the short-circuit current depends on the value of parasitic capacitances between the cable phase cores and screen. Its maximum values result from Eq. (4). With the amplitude modulation factor close to 0, it will reach the maximum value because the inverter CM voltage takes the shape of phase voltage (has rectangular waveform, amplitude equal to 280 V and frequency is 5 kHz). The touch of an ungrounded cable screen with the 3 $\times$ 20 nF parasitic capacitances taken over in the model is as dangerous as the direct touch of the inverter phase voltage (the parasitic capacitance impedance is negligible). In Fig. 5 the $R_{h1}$ resistor means a human touch directly to the wire of inverter phase voltage, while $R_{h2}$ means a human touch of ungrounded cable screen.

**Figure 7:**
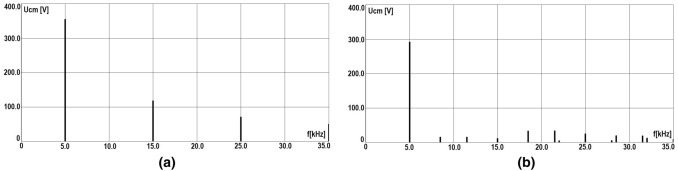
Amplitude spectrum of CM voltage for modulation factor: (**a**) $\hbox {M}=0$, (**b**) $\hbox {M}=0.85$.

Figure 6 shows the waveforms of voltages and ground currents obtained in simulation tests at the occurrence of a ground fault flowing via 1 k$\Omega$ resistor. Figures 6a,b show the short-circuit current waveform with ungrounded cable screen with $\hbox {M} = 0$ and $\hbox {M} = 0.85$, respectively. The obtained waveforms of short-circuit currents indicate that the dominant harmonic amplitude value is 5 kHz in both cases (Fig. 7a,b). Hence, the use of RCD on the power supply of the drive VFC will not work properly when these currents will flow via RCD^22^.

Based on the frequency analysis of the inverter CM voltage (VM5) in Fig. 5, the amplitude spectra of the harmonics were obtained, as shown in Fig. 7a,b. They show that:

- the basic harmonic of the inverter phase voltage with the frequency $f_{c} = 5 \,\hbox {kHz}$ has greater amplitude at the modulation factor $\hbox {M} = 0$ than at $\hbox {M} = 0.85$ (Fig. 7a),
- for the modulation factor M greater than 0, the harmonics of sidebands related to the frequency of the modulating sinusoid will appear, distributed around the even and odd multiple of the modulated frequency $f_{c}$ (Fig. 7b).

## Results

**Figure 8:**
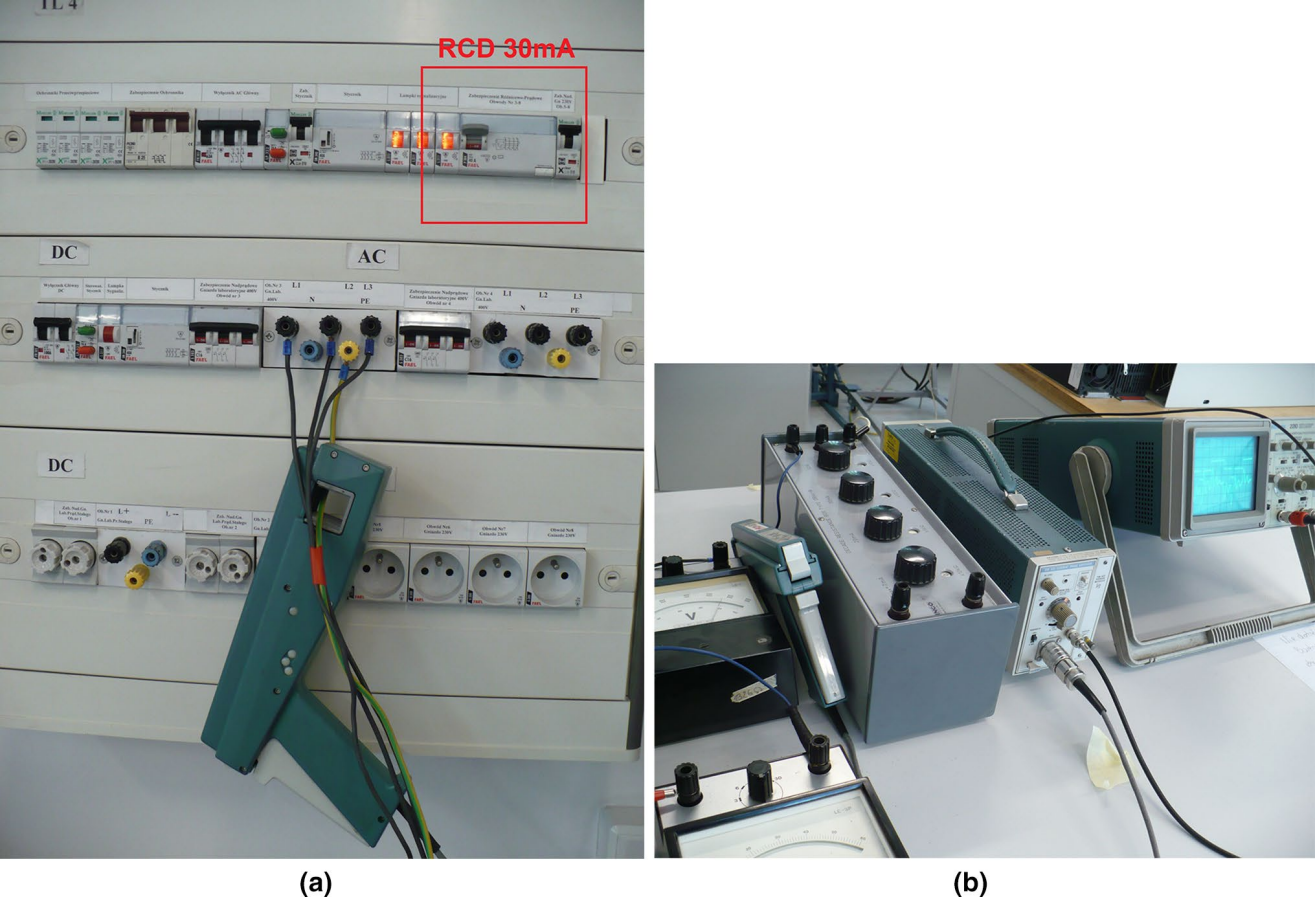
Laboratory stand for short ground current flowing via the human body caused by inverter phase voltage and CM voltage: (**a**) current probe measure of leakage current in PE wire in RCD of drive VFC’s power system, (**b**) measuring of leakage current on an equivalent resistance of the human body 1 k$\Omega$.

Figures 8a,b show the test stand for measuring ground fault currents in the event of a ground fault through a resistance of 1 k$\Omega$ for the following cases:

- shorting of the phase voltage of the inverter,
- shorting of the ungrounded screen of the three-phase cable between the inverter and the rectifier.

The basic elements of the test stand are an oscilloscope set with a 1:100 voltage probe and a current probe with an amplifier, and an adjustable resistor with a range from 0 to 10 k$\Omega$.

**Figure 9:**
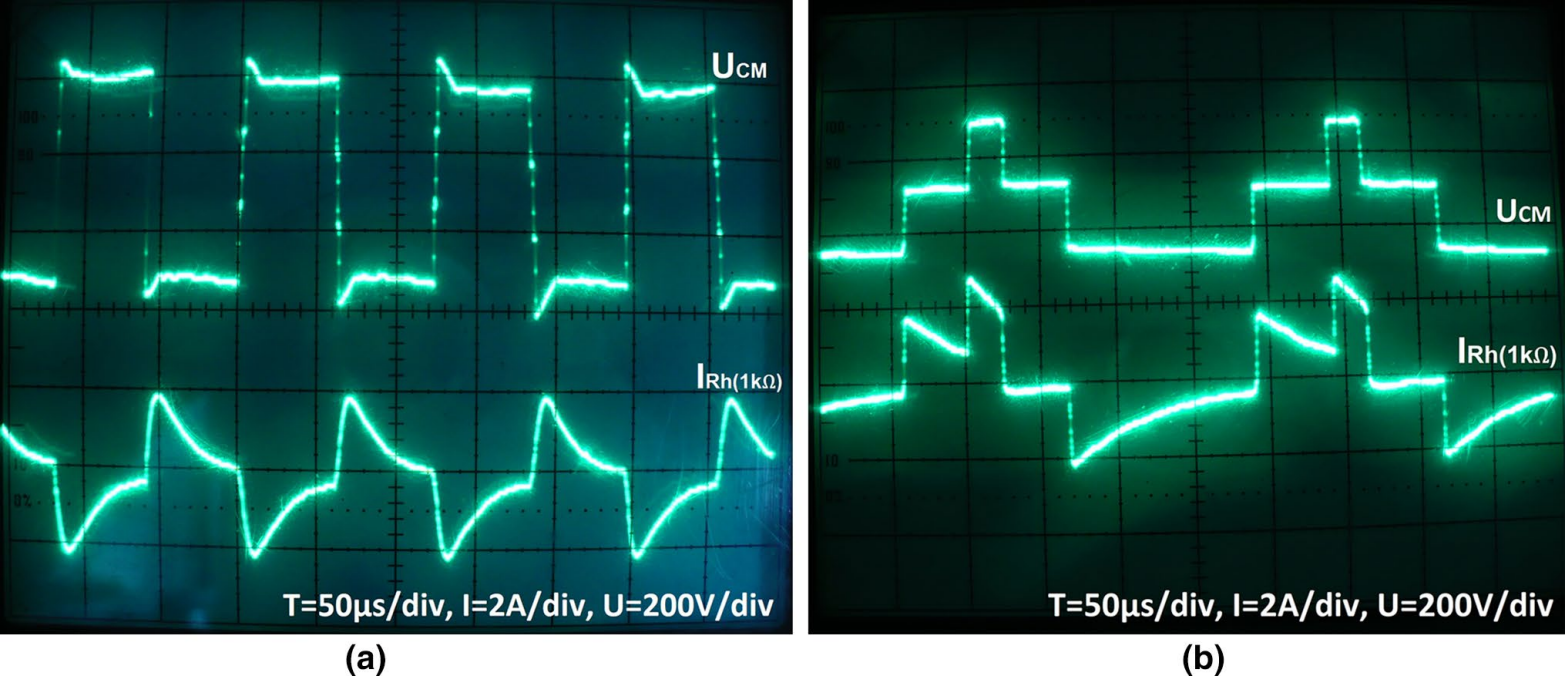
Waveforms of ground fault CM currents at ground resistance 1 k$\Omega$ while: (**a**) CM voltage and CM current for M $\approx$ 0, (**b**) CM voltage and CM current for M = 0.85 (the inverter output basic harmonic frequency is 35 Hz).

The ground current waveform obtained on the laboratory stand at the occurrence of a resistance short-circuit with phase voltage is shown in Fig. 9a. The resistive short-circuit in the ungrounded screen of the three-phase cable (inverter CM voltage) is presented in Fig. 9b. The tests were performed at an output voltage frequency equal to 35 Hz. The currents waveforms of the resistive ground fault at the frequency of 500 Hz are similar, but they are not included in this study. The waveforms of resistive ground fault currents obtained in the experimental tests fully confirm the results from the simulation tests.

## Methods for minimizing the capacitive ground leakage currents

**Figure 10:**
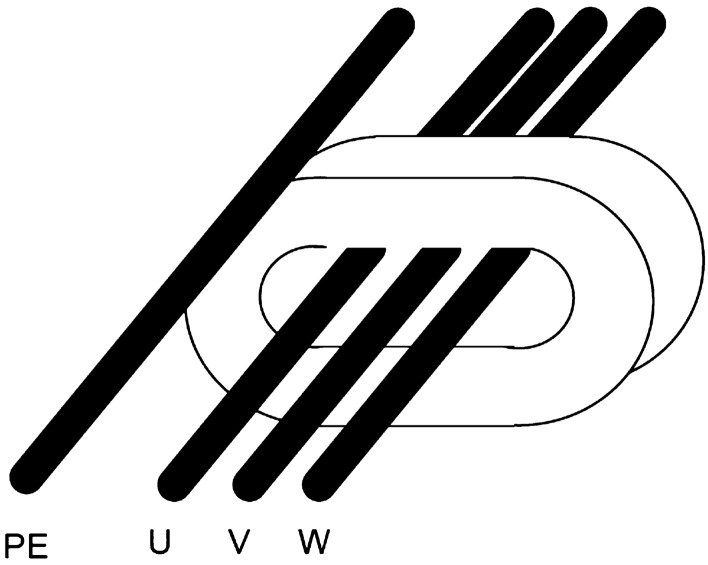
Scheme of special nanocrystalline magnetic cores as a CM filter.

Until now, there are no RCDs correctly operating in the environment of high-frequency ground leakage currents caused by the inverter CM voltage in the industrial offer^14^. The galvanic connection of the PE protective conductor with the common point of the transformer of the TN system supplying the converter produces a low-impedance electric circuit for the ground leakage current. For this reason, the EMC filters built into the converters have a limited ability to absorb the capacitive leakage current caused by the inverter CM voltage^23^. To limit the capacitive leakage current authors propose using screened cable connecting the VFC with the rectifier with reduced parasitic capacitance to ground (in comparison to traditional cables) and CM filter with the special nanocrystalline magnetic cores^24^, as shows Fig. 10.

**Figure 11:**
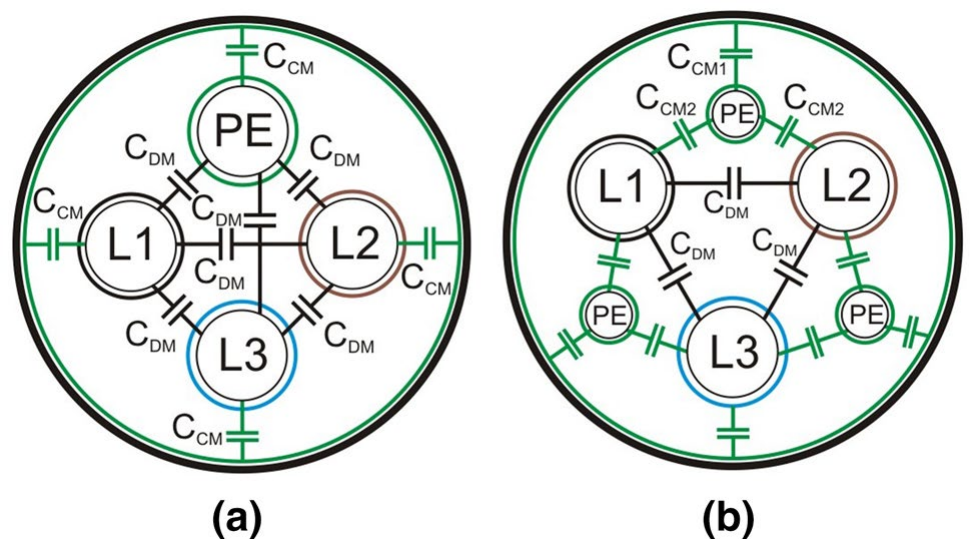
Cross-section of 3-phase screened cables with green marked parasitic line-to-line capacities $C_{DM}$ and parasitic ground leakage capacities $C_{CM}$ in: (**a**) with 1 PE wire, (**b**) with 3 PE wires.

Figure 11 show three-phase screened cables: a traditional one—Fig. 11a and dedicated to connect the inverter with loads (e.g. motors, rectifiers) - Fig. 11b. Based on the catalogue data of screened cable manufacturers^25^, it should be stated that the values of the parasitic ground capacitances of the screened traditional cable are nearly 10 times higher than for the screened cable dedicated to inverters. It is possible to reduce high-frequency leakage currents from the inverter wiring using dedicated cables by nearly 10 times. This reduction is essential to ensure the proper operation of the RCD under normal inverter operating conditions.

The occurrence of a resistive ground fault in the inverter phase conductor will result in a high-frequency short-circuit current that will flow via RCD. Often, such a short-circuit current will not cause the correct operation of the RCD^14^. The authors suggest using an additional ground leakage current sensor in the inverter electric shock protection system (e.g. in PE wire) to stop the inverter operation at the set ground current values, e.g. in the range of 100–300 mA. The proposed range will enable the converter to operate under normal conditions and will stop it, e.g. when directly touching the phase conductor (e.g. 560 V : $2 = 280 \,\hbox {V}$, 280 V : 1 k$\Omega = 280 \,\hbox {mA}$).

**Figure 12:**
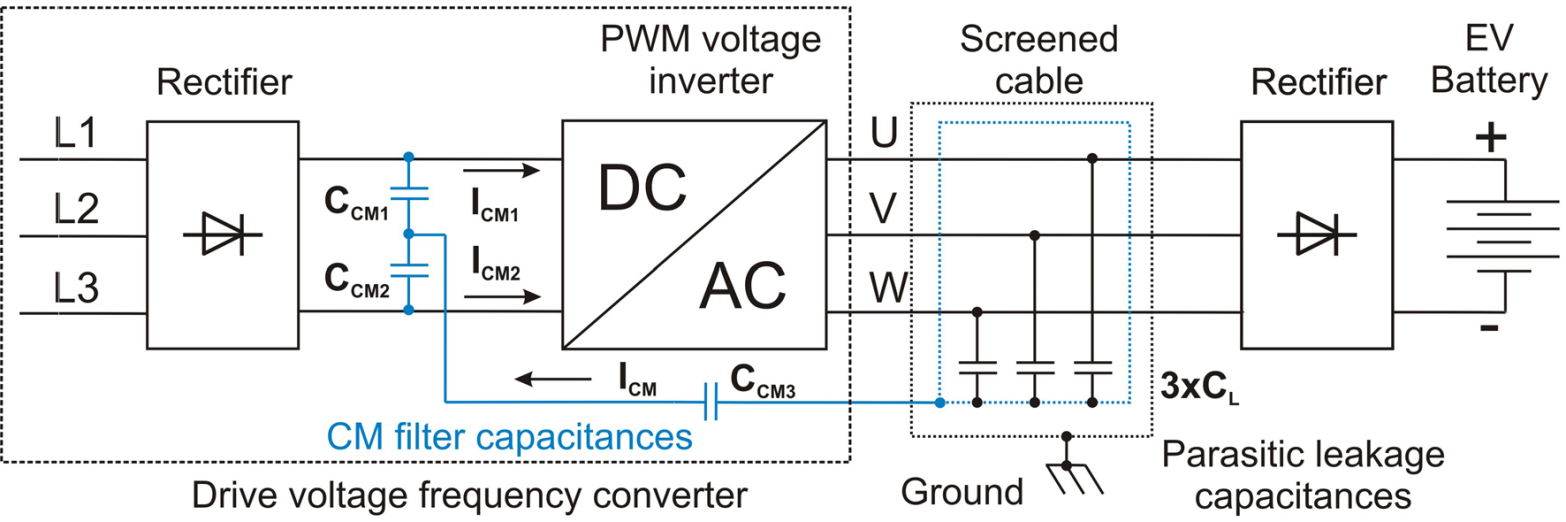
Proposed dedicated circuit for the CM current of the PWM inverter, isolated from the PE protection system.

For long shielded motor cables, i.e. with significant values of the parasitic capacitance between the phase conductors and the cable screen, it may be necessary to eliminate the high-frequency leakage current from the PE protection system. Then the authors propose to create an electric circuit for the capacitive leakage current generated by the CM voltage as shown in Fig. 12^7^. This solution with the additional circuit for CM current allows for the complete elimination of this current from the transformer and RCD. Depending on the construction of the PWM inverter, there may be a need to use additional phase inductances on the AC side of the inverter. They are used to limit the phase-to-phase leakage current generated by the differential-mode (DM) voltage^6^. This means the limitation of DM voltage disturbances of the PWM inverter using an LC sinusoidal filter.

The use of a capacitive filter for CM current allows it to be inserted directly into the DC link of the inverter supply. Then the CM current is completely eliminated from the transformer feeding the converter input rectifier and thus does not disturb the RCD (Fig. 4). In this case, the protection against direct human touch with an RCD is effective.

**Figure 13:**
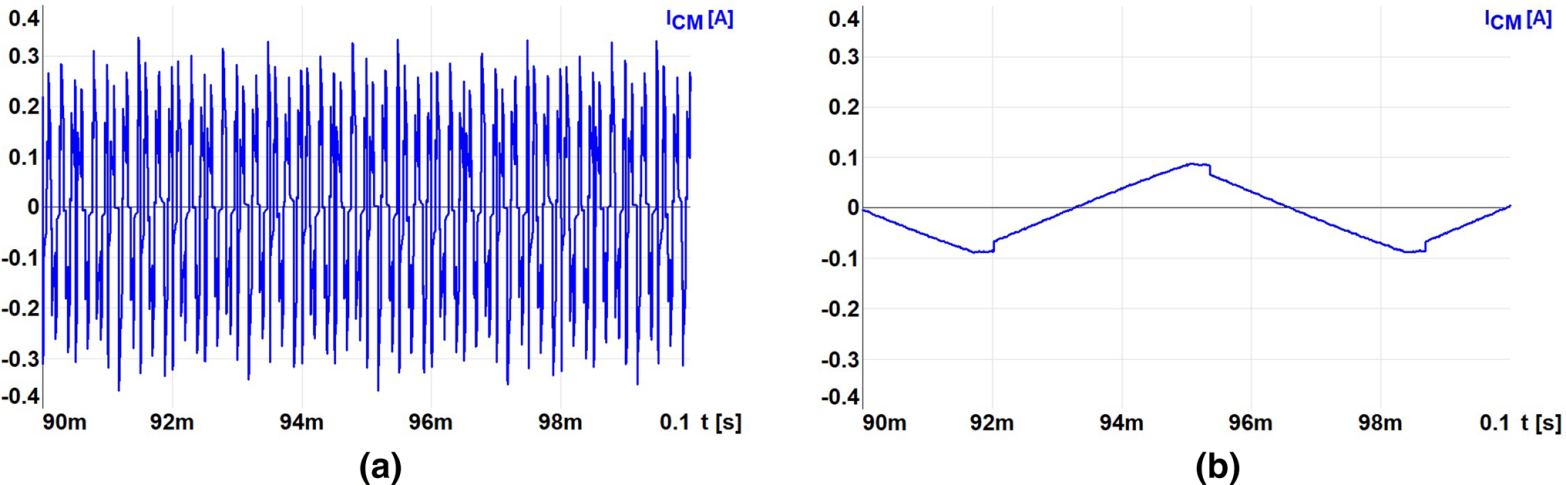
The CM ground leakage current on 1 k$\Omega$ (human touch): (**a**) without CM filter capacitances, (**b**) with CM filter capacitances.

Figure 13 shows the CM current of the PWM inverter with a short-circuit of the ungrounded cable screen to ground via resistance of 1 k$\Omega$. The 1 k$\Omega$ resistor replaces the direct human touch. The simulation tests were performed for two cases without the use of a capacitive CM filter—Fig. 13a and with its use—Fig. 13b. The tests show that the applied capacitive filter (with the parameters: 3 $\times$ $C_L$ = 20 nF, $C_{CM1}$ = $C_{CM2}$ = $C_{CM3}$ = 20 $\upmu$F) completely eliminated the inverter CM current from the PE protection system. Only the rectifier CM current is affected on the PE system, which does not disturb the RCD operation. Such a capacitive filter was added to the model in Fig. 5 and the inverter CM current in the transformer ground conductor was tested.

## Conclusions

The conducted tests of tripping of the AC residual current device with the nominal differential current $I_{\triangle n} = 30 \,\hbox {mA}$ showed that it is insensitive to residual currents with frequency $f_{c}$, regardless of the value of the modulation factor M of the PWM inverter. Measurements of the RCD tripping were performed at the stationary electrical switching station equipped with an RCD type AC (Fig. 8a), which is the supplementary part of the basic protection system. The selection of an appropriate RCD is a very complex task, because the frequency, amplitude, and effective values of the basic harmonic of the resistive short-circuit current must be taken into account. Manufacturers do not specify the properties of RCDs for high-frequency distorted currents.

The conducted tests showed the lack of effective operation of tested RCD in the presence of a high-frequency short-circuit current of the phase voltage and CM voltage of the inverter. According to the authors, the RCD accidental tripping on the Ferranti coil disqualifies it as a supplementary electric shock and fire protection in powering drive VFCs used in the EV battery charging stations and motor drives. To ensure the correct operation of the RCD protection, it is necessary to eliminate the ground leakage current CM of the inverter flowing into the transformer (Fig. 4).

Lowering the value of the ground leakage currents using dedicated screened cables and CM filters enables the correct operation of the RCD installed on the power supply of the converter with the voltage inverter. The inverter CM current filter proposed by the authors (Fig. 12) enables the correct operation of RCD protection in the EV battery charger systems with a PWM inverter.

The occurrence of a resistive ground fault (human touch) should be clearly identified by RCD that will stop the PWM inverter operation immediately if the ground CM current exceeds the allowed value.
